# Environmental exposures and atopic dermatitis: an umbrella review of systematic reviews and meta-analyses

**DOI:** 10.3389/fpubh.2026.1834261

**Published:** 2026-05-15

**Authors:** Sijia Chen, Shipeng Zhang, Wenjing Jiang, Rui Zhou, Qingjia Gu

**Affiliations:** 1 School of Medical and Life Sciences, Chengdu University of Traditional Chinese Medicine, Chengdu, China; 2 Department of ENT, Sichuan Provincial People's Hospital, University of Electronic Science and Technology of China, Chengdu, China

**Keywords:** air pollution, atopic dermatitis, environmental exposure, meta-analysis, smoking, systematic review

## Abstract

**Background:**

Atopic dermatitis (AD) is a common chronic inflammatory skin disorder with a substantial global burden. Although multiple studies have investigated the impact of environmental exposures on AD, a comprehensive review integrating children, adults, and mixed-age populations is lacking. This study aimed to synthesise current evidence on environmental risk factors for AD and examine age-specific susceptibility.

**Methods:**

We systematically searched PubMed, Embase, and Web of Science up to March 2026 for systematic reviews and meta-analyses on environmental exposures and AD risk. Exposures were categorised as: microbe- and immune-related factors (antibiotics and helminth infections), environmental pollution and chemicals (air pollution, per- and polyfluoroalkyl substances (PFAS), heavy metals, pesticides), and lifestyle or residential factors (active and passive smoking, pet exposure, urban environment, light at night, indoor fuel use). Eligible studies reported pooled effect estimates including odds ratios (ORs), relative risks (RRs), or hazard ratios (HRs) with 95% confidence intervals (CIs), or clear directional associations. Data extraction prioritised fully adjusted estimates, dose–response relationships, and age-stratified findings. Methodological quality was assessed using A Measurement Tool to Assess Systematic Reviews 2 (AMSTAR 2). Evidence synthesis focused on effect direction, statistical significance, heterogeneity (I^2^), and overlap of primary studies, without recalculating original estimates.

**Results:**

Twenty systematic reviews and meta-analyses were included. In children, antibiotic exposure and both active and passive smoking were consistently associated with increased AD risk, while prenatal exposure to PM₂.₅ and nitrogen dioxide (NO₂) also elevated risk. In adults, smoking and air pollution showed stable positive associations. In mixed-age populations, PM₁₀ exposure and urban residence were linked to higher AD risk. Evidence for other exposures, including PFAS, heavy metals, pesticides, helminth infection, pet exposure, light at night, and indoor fuel use, remained limited or inconsistent.

**Conclusion:**

Antibiotic exposure, smoking, and air pollution are the most robust environmental risk factors for AD across age groups, whereas other exposures require further investigation. Targeted interventions and environmental management may contribute to AD prevention and control.

## Introduction

Atopic dermatitis (AD) is a common chronic inflammatory skin disorder characterised primarily by disruption of the epidermal barrier and dysregulation of immune homeostasis (1). The onset and progression of AD are shaped by a constellation of factors, most notably genetic susceptibility, environmental exposures and immune perturbations (2). Although its prevalence varies across countries and regions, the overall burden of AD has continued to rise in many parts of the world (3, 4). Current estimates indicate that prevalence reaches as high as 20% in children and 3% in adults, with more than 200 million individuals affected globally (4). Importantly, AD imposes substantial physiological and psychological harm on patients (5, 6), while also generating a considerable economic burden worldwide (7, 8). Elucidating modifiable environmental risk factors, therefore, is of considerable public health importance.

The aetiology of AD is inherently multifactorial, with genetic susceptibility, immune dysregulation and environmental exposures acting in concert to shape disease onset and progression (9). Although heritable factors are central to disease susceptibility, the rapid rise in incidence observed in recent decades suggests that environmental influences may represent key external determinants underpinning the growing burden of disease. Previous investigations have explored the relationship between environmental factors and AD, and several systematic reviews and meta-analyses have addressed specific exposures, including environmental pollutants, air pollutants (10) and tobacco smoke (11). Available evidence indicates that these exposures may contribute to disease development and progression by provoking oxidative stress, compromising epidermal barrier integrity, or modulating immune responses (12). Nevertheless, important gaps remain in the current evidence base. Most existing systematic reviews have focused on single exposures, with limited efforts to integrate findings across exposure categories. At the same time, age-specific differences in environmental risk have not been systematically compared. Moreover, comprehensive evaluations of evidence quality, risk of bias and consistency across findings remain relatively scarce, thereby constraining robust inference regarding the strength of associations and their potential causal relevance.

To address these gaps, we performed an umbrella review of published systematic reviews and meta-analyses examining the association between environmental exposures and AD. Beyond systematically summarising the direction and magnitude of associations across exposure categories, we compared risk profiles across different age groups and performed a stratified appraisal of methodological quality and potential bias. Through this integrative and hierarchical analytical framework, we catalogued the environmental factors examined in existing meta-analyses, assessed their methodological robustness, interrogated potential sources of bias, and identified which associations are supported by the strongest epidemiological evidence. Collectively, these efforts provide an evidence-based foundation for advancing aetiological research and informing preventive strategies for AD.

## Methods

### Search strategy and study selection

This umbrella review was conducted and reported in accordance with the Preferred Reporting Items for Systematic Reviews and Meta-Analyses (PRISMA) 2020 statement. The PRISMA 2020 Checklist is provided in the Supplementary material. Relevant publications were identified using a search strategy that combined Medical Subject Headings (MeSH) and non-MeSH free-text terms for environmental exposures—including antibiotics, smoking, air pollution, helminth infections, per- and polyfluoroalkyl substances (PFAS), heavy metals, pesticides, pet exposure, residential environment, light at night and indoor fuel use—and for outcomes related to atopic dermatitis. Detailed search strategies are provided in the Supplementary Table 1.

### Definition and classification of environmental exposures

In this umbrella review, environmental exposures were broadly defined as external, non-genetic factors arising from the physical, chemical, biological, or lifestyle/residential environment that may influence the development or exacerbation of atopic dermatitis. The exposure categories were determined *a priori* based on a preliminary scoping review of published systematic reviews and meta-analyses, together with MeSH terms and free-text terms identified from database searches. Specifically, MeSH terms were identified from PubMed where available, and non-MeSH free-text terms were added for exposures that are not consistently indexed by MeSH, such as light at night, indoor fuel use, specific air pollutants, PFAS compounds, and heavy metals. Antibiotics were included as external biological exposures because of their potential to modify the microbiota and immune development, whereas light at night was classified as a physical/lifestyle-related exposure because of its potential influence on circadian regulation. Both indoor and outdoor exposures were considered eligible if they were evaluated in relation to AD risk. Indoor exposures included pet exposure, light at night, indoor fuel use, and passive smoking, whereas outdoor exposures included ambient air pollution, pesticides, heavy metals, and urban environment. Both prenatal and postnatal environmental exposures were considered eligible, provided that they were assessed in relation to the risk of AD. Prenatal exposures referred to maternal or in utero environmental exposures evaluated in relation to the subsequent development of AD in offspring. Eligible exposures included, but were not limited to, antibiotics, smoking, air pollutants, helminth infections, PFAS, heavy metals, pesticides, pet exposure, residential environmental factors, light at night, and indoor fuel use.

Eligible studies were systematic reviews or meta-analyses based on observational studies in human populations, including cohort, case–control and cross-sectional designs, that explicitly evaluated the association between environmental exposures and the risk of AD. Reviews were required to report pooled effect estimates, including odds ratios (ORs), relative risks (RRs) or hazard ratios (HRs), together with 95% confidence intervals (CIs), or at minimum to provide a clear direction of association. No age restrictions were imposed; reviews conducted in children, adults or mixed-age populations were all considered eligible. Where age-stratified results were available in the original studies, data for children and adults were extracted separately. We excluded narrative or non-systematic reviews, animal or mechanistic studies, articles that failed to clearly describe the research question, search strategy, databases searched, inclusion and exclusion criteria, or study selection procedures, as well as duplicate publications, studies with incomplete data, and reviews in which AD was not the primary outcome. No predefined restriction was imposed on exposure lag period or latency interval, because the included reviews differed in how they defined exposure windows and follow-up periods. Where available, time-specific exposure results, such as prenatal, early-life, childhood, short-term, or long-term exposure, were extracted and interpreted according to the definitions used in the original systematic reviews or meta-analyses. Accordingly, the synthesised evidence was not limited to cross-sectional data, but may reflect a mixture of longitudinal and cross-sectional associations, depending on the design of the primary studies included in each review.

Two reviewers (GQJ and ZSP) independently screened titles and abstracts and subsequently assessed the full texts according to the predefined inclusion and exclusion criteria. Any disagreements were resolved through discussion between the two reviewers. For studies with unclear eligibility, Supplementary material were further examined, and, where necessary, the original authors were contacted for clarification.

During data extraction, priority was given to the principal pooled effect estimates reported in each meta-analysis, together with their corresponding 95% CIs. The concepts of heterogeneity, small-study effects, and methods to assess them are discussed in Supplementary Tables 2–4. We included studies reporting different measures of OR, RR and HR. The associated *p* values and heterogeneity estimates (I^2^) were also recorded. When multiple analytical models were presented within the same review, the estimate derived from the model with the most comprehensive adjustment for potential confounders was preferentially retained as the primary reference. For studies reporting either fixed-effect or random-effects models, the original estimates provided by the authors were preserved and interpreted in light of the reported degree of heterogeneity. An I^2^ value greater than 50% was considered indicative of moderate to substantial between-study heterogeneity and was taken into account in the interpretation and discussion of the findings. Different effect measures, including ORs, RRs and HRs, were described in a unified manner when the direction of association was consistent. Given that ORs and RRs are often numerically comparable when the outcome is relatively uncommon, the present review focused primarily on the direction and statistical significance of associations rather than undertaking additional conversion or re-pooling of estimates. For dose-specific or multi-category exposure results, priority was given to the overall comparison reported by the original authors; where dose–response analyses were available, the direction and statistical significance of the observed trend were additionally documented.

Given that this study was designed as an overview of reviews, no re-pooling of original individual studies or recalculation of effect estimates was undertaken. Evidence synthesis was based on the pooled estimates reported in previous meta-analyses, and the consistency of associations was appraised by comparing the direction of effect, statistical significance and the degree of heterogeneity across reviews. In interpreting the findings, we further considered the potential overlap of primary studies across different reviews and evaluated the robustness of the evidence accordingly.

### Quality assessment and evidence synthesis

The methodological quality of the included systematic reviews and meta-analyses was assessed using A Measurement Tool to Assess Systematic Reviews 2 (AMSTAR 2). This instrument comprises 16 appraisal items, of which 7 are considered critical domains, addressing key aspects such as protocol registration, the adequacy of the literature search strategy, assessment of risk of bias in the included studies, and consideration of publication bias. Quality assessment was conducted independently by two investigators, and any discrepancies were resolved through discussion until consensus was reached. In accordance with the AMSTAR 2 guidance, the overall methodological quality of each review was classified as high, moderate, low or critically low. The results of the quality appraisal were subsequently taken into account in the synthesis and interpretation of the evidence. The detailed AMSTAR 2 assessment is presented in Supplementary Table 5. To provide a more structured and transparent assessment of evidence certainty, we additionally applied a simplified Grading of Recommendations Assessment, Development and Evaluation (GRADE)-informed framework across the major exposure categories in children, adults, and mixed-age populations, considering risk of bias, inconsistency, indirectness, imprecision, publication bias, and, where available, dose–response relationship. This approach was adopted in the context of an umbrella review, where full implementation of the GRADE framework is often not feasible.

For evidence synthesis, no new statistical pooling or recalculation of effect estimates was undertaken; instead, comparisons were based on the findings reported in previous meta-analyses. Judgements regarding the associations between different environmental exposures and AD were made by jointly considering statistical significance (*p* < 0.05), the magnitude of the effect estimates and their confidence intervals, the degree of between-study heterogeneity, and the presence of publication bias, while also taking into account the methodological quality of the included reviews. Where substantial discrepancies were observed across findings, or where methodological quality was limited, the results were interpreted with appropriate caution and the attendant uncertainty was explicitly acknowledged. If conclusions across reviews were inconsistent or lacked statistical support, the available evidence was considered insufficient to support a definitive conclusion. The results of the simplified GRADE-informed certainty assessment are presented in Supplementary Table 6. The main abbreviations used in this study and their corresponding full terms are provided in the Supplementary Table 7.

## Results

**Figure 1:**
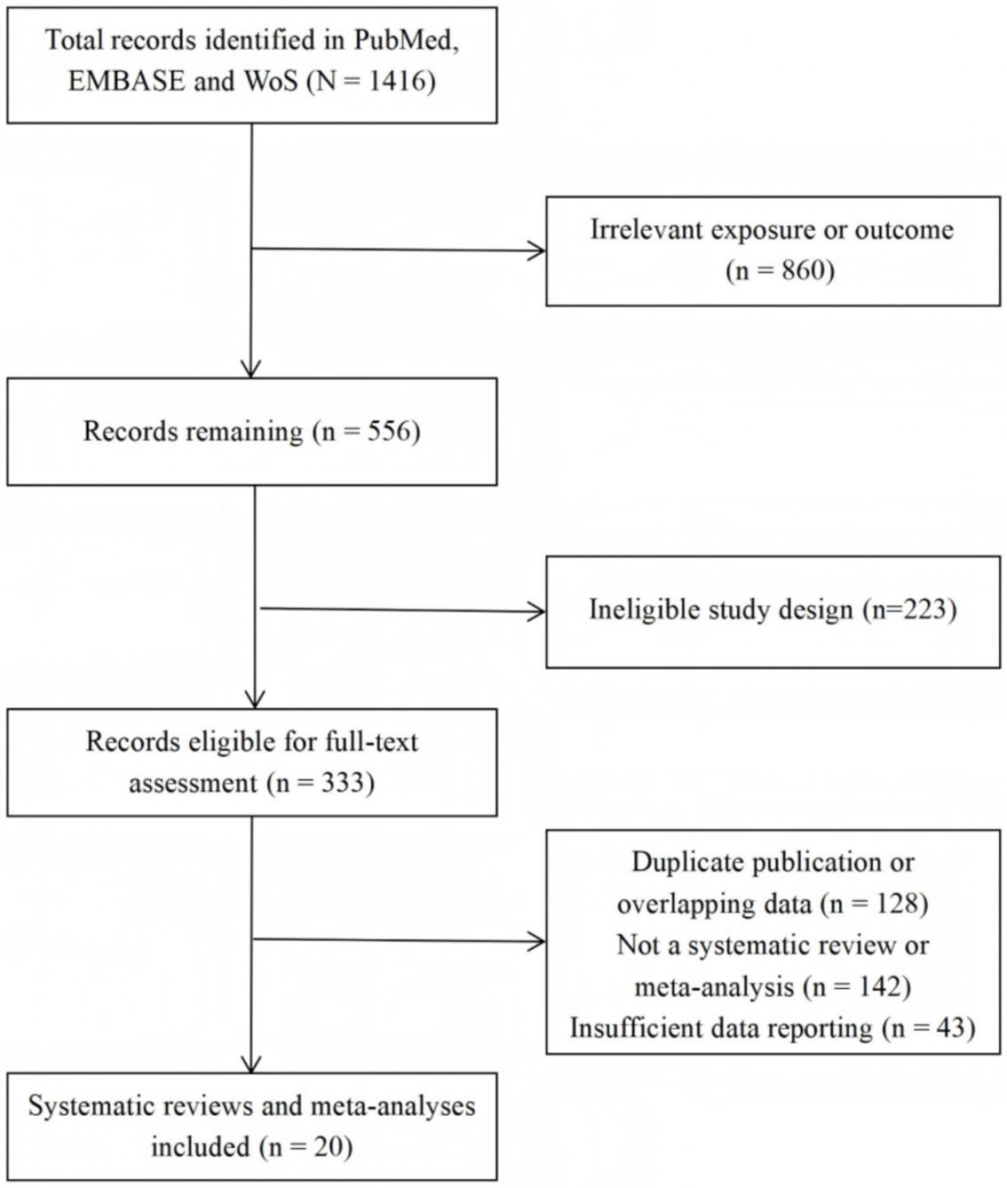
The document screening flow chart was included.

A total of 1,416 records were identified through the literature search (Figure 1). After screening titles and abstracts, 1,083 clearly irrelevant articles were excluded, leaving 333 records for full-text assessment. Following full-text review, 313 articles were further excluded, primarily because of duplicate publication or overlapping data, failure to meet the definition of a systematic review or meta-analysis, or insufficient reporting of relevant data. Ultimately, 20 systematic reviews and meta-analyses were included. The included reviews encompassed a broad range of environmental exposures, including antibiotics (*n* = 3) and smoking (*n* = 3); air pollution (*n* = 3), helminth infection (*n* = 2), PFAS (*n* = 2), and heavy metals (*n* = 2); as well as pesticides (*n* = 1), pet exposure (*n* = 1), urban environment (*n* = 1), light at night (*n* = 1), and indoor fuel use (*n* = 1). Based on their exposure characteristics, these factors were grouped into three broad categories: microbe- and immune-related factors, environmental pollution and chemical exposures, and lifestyle and residential environmental factors. The study populations included children, adults and mixed-age populations, with the underlying evidence derived predominantly from observational studies.

### Environmental exposures in children

**Figure 2:**
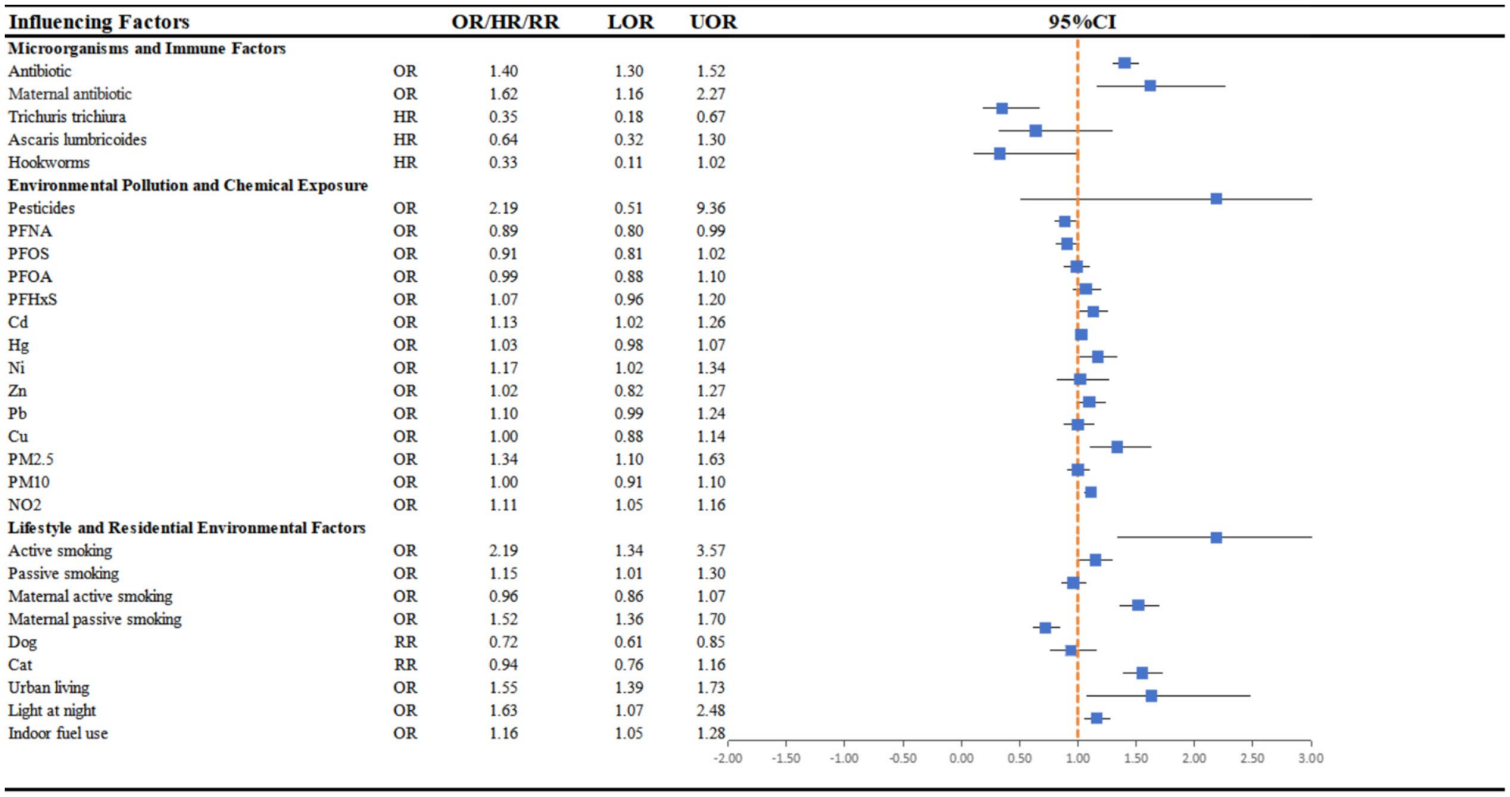
Forest plot: summary effect estimates of meta-analyses reporting associations between atopic dermatitis and factors pertaining to microorganisms and immune factors, environmental pollution and lifestyle and residential environmental factors among children. CI, confidence interval; OR, odds ratio; HR, hazard ratio; RR, relative risk.

A total of 20 systematic reviews and meta-analyses were ultimately included in this umbrella review. Among these, 19 reviews involving children are presented in Figure 2 and Supplementary Table 2. The environmental exposures examined could be broadly classified into three categories: microbe- and immune-related factors, including antibiotics (*n* = 3) (13–15) and helminth infections (*n* = 2) (16, 17); environmental pollution and chemical exposures, including air pollution (*n* = 2) (10, 18), PFAS (*n* = 2) (19, 20), heavy metals (*n* = 2) (2, 5), and pesticides (*n* = 1) (21); and lifestyle and residential environmental factors, including smoking (*n* = 3) (11, 22, 23), pet exposure (*n* = 1) (24), urban environment (*n* = 1) (4), light at night (*n* = 1) (25), and indoor fuel use (*n* = 1) (7). In terms of consistency of evidence, the associations for antibiotics and air pollution were the most consistently supported.

All three meta-analyses on antibiotics indicated that antibiotic exposure during pregnancy or childhood was associated with an increased risk of AD. Likewise, both reviews on air pollution consistently demonstrated that prenatal exposure to PM₂.₅ and NO₂ was significantly associated with a higher risk of AD, whereas the evidence for PM₁₀ remained inconclusive. For smoking, most studies suggested an elevated risk, particularly for active smoking and passive exposure during childhood; by contrast, evidence for active maternal smoking during pregnancy was limited, whereas findings for passive smoking during pregnancy were comparatively consistent. Detailed information on smoking duration or number of years of smoking was not consistently reported in the included meta-analyses.

In contrast, the evidence for PFAS (*n* = 2), heavy metals (*n* = 2), and pesticides (*n* = 1), appeared more substance-specific or directionally heterogeneous. The two reviews on helminth infections suggested that certain early-life infections might confer a protective effect, although the overall evidence remained limited. Among lifestyle-related factors, pet exposure (*n* = 1) was associated with a potential protective effect. Urban environment, indoor fuel use, and light at night (*n* = 1) were all reported to show a trend toward increased risk, although the number of available studies was small. Taken together, the strength and consistency of evidence linking environmental exposures to AD varied substantially across exposure domains. At present, antibiotic use and air pollution exposure emerge as the most consistently supported risk factors, whereas the remaining associations require further investigation before firmer conclusions can be drawn.

### Environmental exposures in adults

**Figure 3:**
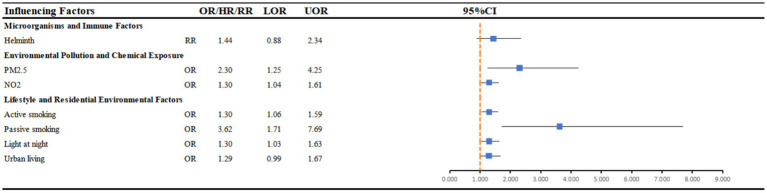
Forest plot: summary effect estimates of meta-analyses reporting associations between atopic dermatitis and factors pertaining to microorganisms and immune factors, environmental pollution and lifestyle and residential environmental factors among adults. CI, confidence interval; OR, odds ratio; HR, hazard ratio; RR, relative risk.

Figure 3 summarises seven systematic reviews and meta-analyses examining a range of environmental exposures during adulthood in relation to AD risk (see Supplementary Table 3). Current evidence indicates that the associations for smoking (*n* = 2) and air pollution (*n* = 2) are the most consistent. Both meta-analyses on smoking demonstrated that active smoking in adulthood was associated with an increased risk of AD, with passive smoking potentially conferring an even greater risk. Similarly, the two available reviews on air pollution reported a concordant positive association between exposure to PM₂.₅ and NO₂ and elevated AD risk.

By contrast, light at night (*n* = 1) was suggestive of an increased risk, although the observed effect appeared comparatively modest, whereas urban environment (*n* = 1) and helminth infection (*n* = 1) have not, thus far, shown statistically significant associations. Taken together, the most consistent evidence in adult populations centres on smoking and air pollution, while the roles of other environmental factors remain to be clarified through further investigation.

### Environmental exposures in mixed-age populations

**Figure 4:**
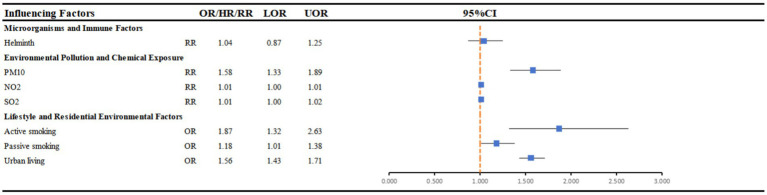
Forest plot: summary effect estimates of meta-analyses reporting associations between atopic dermatitis and factors pertaining to microorganisms and immune factors, environmental pollution, and lifestyle and residential environmental factors across all age groups. CI, confidence interval; OR, odds ratio; HR, hazard ratio; RR, relative risk.

Figure 4 includes five systematic reviews and meta-analyses evaluating the associations between different environmental exposures and AD risk (see Supplementary Table 4). In these studies, the included populations were generally not stratified by age, and participants across a broad age range were analysed together, with limited consistency in the definition of age groups across reviews. Overall, the evidence for smoking (*n* = 2) was relatively consistent. Both meta-analyses indicated that active smoking was associated with an increased risk of AD, and household passive smoking likewise showed a concordant positive association (11, 22). With respect to air pollution (*n* = 1), one meta-analysis assessed the effects of both short-term and long-term exposure on AD risk and demonstrated that long-term exposure to PM₁₀, as well as short-term exposure to PM₁₀, NO₂ and SO₂, was associated with an increased risk of AD or eczema, with a consistent direction of effect across short- and long-term exposure windows.

Urban residence (*n* = 1) was reported in one meta-analysis to be associated with a higher risk of AD (4), although the limited number of available studies warrants cautious interpretation. By contrast, helminth infection (*n* = 1) did not show a significant overall association. Taken together, the most consistent evidence continues to centre on smoking and air pollution, whereas support for other environmental factors remains limited.

A simplified GRADE-informed framework was further applied to assess the certainty of evidence across the major exposure categories (Supplementary Table 6). Overall, the certainty of evidence was low to very low, reflecting the observational nature of the underlying evidence and limitations related to risk of bias, inconsistency, imprecision, and limited dose–response information. In children, low-certainty evidence was observed for antibiotic exposure and air pollution, whereas evidence for helminth infections, PFAS, heavy metals, smoking, pet exposure, urban living, light at night, and indoor fuel use was rated as very low certainty. In adults, the evidence for light at night, smoking, urban living, helminth infections, and air pollution was generally rated as very low certainty, largely owing to inconsistency, imprecision, or limited exposure-specific evidence. In mixed-age populations, urban living was supported by low-certainty evidence, whereas smoking, helminth infections, and air pollution were rated as very low certainty. Dose–response evidence was limited overall and was mainly observed for selected air pollution-related exposures.

## Discussion

By systematically comparing the evidence linking diverse environmental exposures to AD, this study identified multiple exposure-related signals that were either consistent or directionally coherent in relation to disease risk. Overall, pollution-related exposures tended to be associated with an increased risk of AD, whereas certain microbe-related exposures appeared to be linked to a reduced risk. The strength of these associations varied across life stages, with findings for exposures during childhood and pregnancy appearing comparatively more stable. Collectively, these observations suggest that the environmental determinants of AD cannot be explained by any single factor in isolation, but instead likely reflect the combined influence of multiple external exposures, potentially involving immune development, epidermal barrier function, and circadian regulation.

### Differences in environmental associations across life stages

The age-stratified analyses in this study indicate that environmental associations with AD are not uniform across the life course. Compared with adulthood, pregnancy and childhood were more likely to exhibit patterns of increased risk, whereas findings in adults showed greater heterogeneity. In children, the association between antibiotic exposure and elevated AD risk was relatively consistent, with prenatal exposure appearing to exert a stronger effect than postnatal exposure, a pattern broadly consistent with previous birth cohort studies (13–15). Perturbations in the early-life microbiota or microbial exposure profile have been proposed to disrupt immune development, thereby increasing susceptibility to allergic disease (26, 27). However, the available reviews provided limited detail on antibiotic classes and pregnancy-specific exposure timing. Prenatal exposure to PM₂.₅ and NO₂ showed a similar pattern, and previous studies have suggested that maternal exposure to air pollution may heighten the risk of childhood AD by altering fetal immune development (10). Urban residence also tended to be associated with increased risk in children, whereas no comparable consistency was observed in adults; this discrepancy may reflect the greater vulnerability of the developing immune system to environmental insults during early life. Nevertheless, adulthood was not devoid of meaningful associations. PM₂.₅ remained strongly associated with adult-onset AD. Previous studies have likewise indicated that air pollution is more frequently linked to disease activity or symptom exacerbation in adult AD than to incident disease, a pattern broadly aligned with the findings of the present review (1, 18). This interpretation is further supported by a recent meta-analysis, which reported that environmental factors such as air pollution, temperature extremes, and humidity were associated with increased AD burden, particularly in terms of disease severity and health care utilisation in adults, rather than incident disease alone (28). This observation is more consistent with the notion that sustained exposure contributes to disease maintenance or aggravation rather than serving as the initial trigger. Smoking showed a concordant direction of association across age groups, in line with previous reports, suggesting that certain environmental factors may exert persistent effects throughout different stages of life (11, 22). Taken together, the associations observed during pregnancy and early childhood appeared more consistent, indicating that responses to environmental exposures may vary across the life course.

### Potential directions of effect of different environmental factors

The age-related differences observed above may, at least in part, reflect the distinct biological mechanisms through which different environmental factors exert their effects. To better interpret these associations, a mechanistic framework is warranted. Broadly, the relevant exposures can be considered within three biological pathways: microbe–immune regulation, pollution-mediated barrier injury, and circadian disruption, referring to disturbances in circadian rhythmicity, such as alterations in sleep–wake cycles and melatonin secretion, which may in turn influence immune responses. Although these factors differ in their proximal characteristics, they converge to some extent in their capacity to amplify oxidative stress, promote immune skewing, and alter barrier function. Such mechanistic overlap may represent a shared biological basis underlying their associations with AD risk.

#### Microbe-related exposures

A range of environmental exposures may influence the development of AD by altering microbial communities and, in turn, modulating immune regulation. Antibiotic use can disrupt the composition of the gut and skin microbiota, interfere with the establishment of immune tolerance, and thereby increase the risk of AD (29, 30). Although such perturbations may be transient, their effects on immune responses and inflammatory processes may nevertheless be sufficient to heighten susceptibility to allergic disease (13, 14). This effect may be particularly relevant when antibiotics are administered during pregnancy, as alterations in the maternal and fetal-associated microbiota could further impair immune tolerance and increase the risk of AD in the offspring (15). However, the included meta-analyses generally evaluated antibiotic exposure as a broad category and did not provide sufficiently detailed information on specific antibiotic classes or types. In addition, the timing of exposure during pregnancy, such as trimester-specific effects, was not consistently reported. As a result, the available evidence does not allow for a more refined interpretation of differential risks, and these aspects warrant further investigation in future studies. By contrast, helminth infections, such as Trichuris trichiura, may be associated with a reduced risk of AD. One study reported that T. trichiura infection during infancy (HR 0.35) might lower the risk of AD through immunomodulatory mechanisms. In addition, pet exposure, particularly exposure to dogs, has been associated with a significantly reduced risk of AD (RR 0.72). The hygiene hypothesis posits that diminished microbial exposure in daily life may constrain normal immune maturation, thereby increasing the risk of allergic disease (31). The potentially protective effect of dog exposure on AD provides further support for this hypothesis.

#### Pollution- and chemical-related exposures

Exposure to air pollution, heavy metals, PFAS, pesticides, and indoor fuels has all been implicated in AD risk. These exposures are thought to contribute to disease development through several interconnected mechanisms, including amplification of oxidative stress, induction of epigenetic alterations, and impairment of epidermal barrier integrity (21). In addition, air pollutants such as polycyclic aromatic hydrocarbons may contribute to AD through activation of the aryl hydrocarbon receptor (AhR) pathway, which has also been implicated in inflammatory signalling and epidermal dysfunction (32). Among them, the evidence for PM₂.₅ is particularly notable. One study reported that each 10 μg/m^3^ increment in prenatal PM₂.₅ exposure was associated with a 34% increase in AD risk, suggesting that maternal exposure may influence fetal immune development through transplacental pathways (10). Likewise, prenatal exposure to NO₂ and to heavy metals such as nickel and cadmium has been associated with an elevated risk of AD in infants and young children, potentially through altered immune responses and enhanced immunoglobulin production (33–36). Chemical constituents in pesticides may also aggravate AD by activating immune pathways, intensifying cutaneous hypersensitivity, or directly compromising skin barrier function (21). By contrast, the findings for PFAS are more complex. Certain compounds, such as perfluorononanoic acid (PFNA), have even been associated with a reduced risk of eczema, although the reasons for this remain unclear, and the influence of residual confounding or selection bias cannot be excluded. Some included studies also suggested that the relationship between environmental exposure and AD risk may involve threshold effects or non-linear exposure–response patterns, rather than a purely linear increase in risk. This appeared particularly relevant for air pollution-related exposures, where risk estimates may vary according to the exposure range and modelling approach used. Overall, pollution-related signals can be observed across multiple life stages, with the evidence for prenatal exposure appearing comparatively more consistent.

#### Lifestyle-related factors

Although the relationship between smoking and AD has not been fully elucidated, existing evidence suggests that active smoking may promote the development of AD by inducing oxidative stress in keratinocytes and disrupting cutaneous lipid homeostasis and barrier integrity (37). AD is also frequently accompanied by psychological distress, and such distress is strongly associated with smoking behaviour, raising the possibility that these factors may act synergistically to exacerbate disease onset and progression (38, 39). Moreover, in adults, smoking is associated not only with an elevated risk of AD but also with a wide range of adverse health outcomes, including cancer and cardiovascular disease (40). By contrast, passive smoking may exert a more pronounced effect in children, potentially owing to the cumulative burden of tobacco smoke exposure. Urban environments, characterised by higher levels of allergens and pollutants, may likewise contribute to AD by compromising epidermal barrier function (41–43), whereas the skin barrier in adults may be more resilient to such insults than that of children. Light at night represents another potentially important lifestyle-related exposure. It may indirectly promote cutaneous allergic responses by altering the expression of circadian genes and suppressing melatonin secretion. Melatonin has been reported to reduce immunoglobulin E (IgE) production and may therefore exert a protective effect in AD, whereas nighttime light exposure could enhance skin allergic responses indirectly through melatonin suppression (44). This effect may be particularly relevant in adolescents, who appear to be more susceptible to circadian disruption (25, 45–47). In addition, the use of indoor fuels, especially in poorly ventilated or enclosed settings, can release airborne pollutants that intensify cutaneous inflammation and further impair barrier function, thereby increasing the risk of AD.

### Strengths and limitations

This study stratified environmental exposures across different stages of the life course and compared the observed associations in children, adults, and mixed-age populations. In contrast to previous reviews that did not distinguish between age groups, this framework revealed that many of the significant associations were concentrated in childhood. By presenting the findings in an age-stratified manner, the analysis more clearly delineates the potential heterogeneity of environmental effects across developmental stages. At the same time, the inclusion of a broad range of environmental exposures allowed for comparison across exposure domains and provided a basis for identifying age-specific priorities for prevention and intervention.

Several limitations should nevertheless be acknowledged. First, exposure assessment methods and classification criteria varied across studies, which may have introduced a degree of measurement error. Second, part of the evidence base was derived from cross-sectional studies, limiting causal inference and precluding full exclusion of reverse causation and residual confounding. In addition, most included studies were conducted in high-income countries, whereas evidence from other regions remains scarce, thereby limiting the generalizability of the findings across diverse populations and settings. Some analyses were also constrained by small sample sizes or missing data, and the relative paucity of null findings raises the possibility of publication bias. Furthermore, to address the need for a structured evaluation of evidence certainty, we incorporated a simplified GRADE-informed framework in the revised analysis. However, given the umbrella review design and the reliance on previously published meta-analyses rather than primary studies, this approach should be interpreted as a structured approximation rather than a full formal GRADE assessment. Based on this simplified GRADE-informed assessment, the overall certainty of evidence across most environmental exposures was rated as low to very low, which may be attributable to heterogeneity across the included studies and the limited number of available studies for certain exposures. Overall, although this study provides a relatively comprehensive synthesis of the available evidence and highlights differences across life stages, the conclusions should be further substantiated by prospective studies and analyses that account for mixed or combined exposures.

## Conclusion

Environmental factors may act through multiple biological pathways to shape the onset and progression of AD. From alterations in the microbial milieu and exposure to pollutants to the influence of lifestyle-related factors, these external determinants are likely to exert differential effects across stages of the life course. Appropriate microbial exposure in early life, together with the judicious use of antibiotics, may play an important protective role in immune development and the establishment of immune tolerance. By contrast, pollutant exposure during pregnancy and early infancy may increase the risk of AD by disrupting epidermal barrier function, activating immune responses, and perturbing immune development. Reducing such exposures during these vulnerable periods should therefore constitute an important target for AD prevention. At the same time, lifestyle-related factors such as smoking and light at night further underscore the importance of behavioural patterns and psychological status in the development of AD. Smoking cessation, healthier behavioural practices, and, in particular, strengthened health interventions for adolescents may help reduce the burden of AD. Taken together, the prevention and management of AD require not only attention to environmental and genetic determinants, but also multidimensional intervention at the level of individual lifestyle. Future research should be expanded across more diverse regions and interpreted in the context of different life stages so as to inform more precise and effective public health strategies.

## Data Availability

The original contributions presented in the study are included in the article/Supplementary material, further inquiries can be directed to the corresponding authors.
