# Supplementary material for: Environmental exposures and atopic dermatitis: an umbrella review of systematic reviews and meta-analyses
Source: Front Public Health. 2026 May 15;14:1834261. doi: 10.3389/fpubh.2026.1834261 (PMC13219279; doi:10.3389/fpubh.2026.1834261)
Supplement: Supplementary file 1 [file Data_Sheet_1.docx]

Search Strategy

Search Strategy Free text search strategy: Initial search date: March 2026

**Supplemental Table 1**: Medical Subject Headings (MeSH) and non-MeSH free-text terms were used to identify relevant publications on the associations between environmental exposures—including antibiotics, smoking, air pollution, helminth infections, per- and polyfluoroalkyl substances (PFAS), heavy metals, pesticides, pet exposure, urban environment, light at night, and indoor fuel use—and atopic dermatitis.

| **Database** | **Step** | **Terms** | **Results** |
| --- | --- | --- | --- |
| **PubMed** | 1 | ("Dermatitis, Atopic"[Mesh]) OR (Atopic Dermatitis[Title/Abstract])) OR (Eczema, Atopic[Title/Abstract])) OR (Atopic Eczema[Title/Abstract])) OR (Neurodermatitis, Atopic[Title/Abstract])) OR (Atopic Neurodermatitis[Title/Abstract])) OR (Neurodermatitis, Disseminated[Title/Abstract])) OR (Disseminated Neurodermatitis[Title/Abstract])) OR (Eczema, Infantile[Title/Abstract])) OR (Infantile Eczema[Title/Abstract]) | 42998 |
|  | 2 | ("Anti-Bacterial Agents"[Mesh]) OR ("Anti-Bacterial Agents"[Title/Abstract] OR "Anti Bacterial Agents"[Title/Abstract] OR "Anti-Bacterial Compounds"[Title/Abstract] OR "Anti Bacterial Compounds"[Title/Abstract] OR "Antibacterial Agents"[Title/Abstract] OR "Anti-Bacterial Agent"[Title/Abstract] OR "Anti Bacterial Agent"[Title/Abstract] OR "Anti-Bacterial Compound"[Title/Abstract] OR "Anti Bacterial Compound"[Title/Abstract] OR "Antibacterial Agent"[Title/Abstract] OR "Antibiotics"[Title/Abstract] OR "Antibiotic"[Title/Abstract] OR "Anti-Mycobacterial Agents"[Title/Abstract] OR "Anti Mycobacterial Agents"[Title/Abstract] OR "Antimycobacterial Agents"[Title/Abstract] OR "Anti-Mycobacterial Agent"[Title/Abstract] OR "Anti Mycobacterial Agent"[Title/Abstract] OR "Antimycobacterial Agent"[Title/Abstract] OR "Bacteriocidal Agents"[Title/Abstract] OR "Bacteriocides"[Title/Abstract] OR "Bacteriocidal Agent"[Title/Abstract] OR "Bacteriocide"[Title/Abstract]) | 791793 |
|  | 3 | ("Smoking"[Mesh]) OR ("Smoking Behaviors"[Title/Abstract] OR "Smoking Behavior"[Title/Abstract] OR "Smoking Habit"[Title/Abstract] OR "Smoking Habits"[Title/Abstract]) | 180587 |
|  | 4 | ("Air Pollution"[Mesh]) OR ("Air Pollution"[Title/Abstract] OR "Air Quality"[Title/Abstract]) | 109586 |
|  | 5 | ("Helminthiasis"[Mesh]) OR ("Helminthiases"[Title/Abstract] OR "Nematomorpha Infection"[Title/Abstract] OR "Nematomorpha Infections"[Title/Abstract]) | 141084 |
|  | 6 | ("Fluorocarbons"[Mesh] ) OR ("Perfluorocarbon"[Title/Abstract] OR "Fluorocarbon"[Title/Abstract] OR "Perfluorocarbons"[Title/Abstract] OR "Polyfluorocarbons"[Title/Abstract] OR "Per- and Polyfluoroalkyl Substances"[Title/Abstract] OR "Per and Polyfluoroalkyl Substances"[Title/Abstract] OR "PFAS"[Title/Abstract] OR "Fluorinated Telomer Alcohols"[Title/Abstract] OR "Fluoro-Telomer Alcohols"[Title/Abstract] OR "Fluoro Telomer Alcohols"[Title/Abstract] OR "Polyfluorinated Telomer Alcohols"[Title/Abstract] OR "Perfluoroalkyl Carboxylates"[Title/Abstract] OR "Perfluoroalkane Sulfonamides"[Title/Abstract] OR "Perfluoroalkyl Ether Carboxylates"[Title/Abstract] OR "Fluorotelomer Phosphate Esters"[Title/Abstract] OR "Fluorocarbon Emulsions"[Title/Abstract] OR ("Fluorocarbon Emulsion"[Title/Abstract] ) OR "Perfluoropolyether Carboxylic Acids"[Title/Abstract]) | 30962 |
|  | 7 | ("Metals, Heavy"[Mesh] )OR ("Heavy Metals"[Title/Abstract] OR "Heavy Metal"[Title/Abstract]) | 735202 |
|  | 8 | ("Pesticides"[Mesh]) OR (Pesticide[Title/Abstract]) | 145050 |
|  | 9 | ("Environment"[Mesh]) OR ("Environments"[Title/Abstract] OR "Environmental Impact"[Title/Abstract] OR "Environmental Impacts"[Title/Abstract]) | 1970081 |
|  | 10 | ("Light"[Mesh]) OR ("Photoradiation"[Title/Abstract] OR "Photoradiations"[Title/Abstract] OR "Visible Light"[Title/Abstract] OR "Visible Radiation"[Title/Abstract] OR "Visible Radiations"[Title/Abstract]) | 369116 |
|  | 11 | ("Fuel Oils"[Mesh]) OR ("Fuel Oil"[Title/Abstract] OR "Fuel Oils"[Title/Abstract]) | 2275 |
|  | 12 | ("Pets"[Mesh]) OR ("Pet"[Title/Abstract] OR "Companion Animals"[Title/Abstract] OR "Companion Animal"[Title/Abstract] OR "Companion Dogs"[Title/Abstract] OR "Companion Dog"[Title/Abstract] OR "Companion Cats"[Title/Abstract] OR "Companion Cat"[Title/Abstract]) | 164822 |
|  | 13 | "Meta-Analysis" [Publication Type] | 235420 |
|  | 14 | #2 OR #3 OR #4 OR #5 OR #6 OR #7 OR #8 OR #9 OR #10 OR #11 OR #12 OR AND #13 | 18439 |
|  | 15 | #1 AND #14 | **57** |
| **Embase** | 1 | 'atopic dermatitis'/exp OR 'atopic constitutional neurodermatitis':ti,ab,kw OR 'atopic eczema':ti,ab,kw OR 'atopic neurodermatitis':ti,ab,kw OR 'coca sulzberger disease':ti,ab,kw OR 'coca sulzberger syndrome':ti,ab,kw OR 'dermatitis, atopic':ti,ab,kw OR 'eczema atopica':ti,ab,kw OR 'eczema endogenous':ti,ab,kw OR 'endogenous eczema':ti,ab,kw OR 'neurodermatitis constitutionalis':ti,ab,kw OR 'neurodermatitis disseminata':ti,ab,kw OR 'neurodermatitis, atopic constitutional':ti,ab,kw OR 'atopic dermatitis':ti,ab,kw | 80144 |
|  | 2 | 'antiinfective agent'/exp OR 'anti bacterial agent':ti,ab,kw OR 'anti bacterial agents':ti,ab,kw OR 'anti infective agents':ti,ab,kw OR 'anti infectives, otic':ti,ab,kw OR 'anti-bacterial agents':ti,ab,kw OR 'anti-infective agents':ti,ab,kw OR 'anti-infectives, otic':ti,ab,kw OR 'antibacterial':ti,ab,kw OR 'antibacterial agent':ti,ab,kw OR 'antibacterial drug':ti,ab,kw OR 'antibacterial soap':ti,ab,kw OR 'antibacterial spectrum':ti,ab,kw OR 'antiinfectives, otic':ti,ab,kw OR 'antimicrobial':ti,ab,kw OR 'antimicrobial agent':ti,ab,kw OR 'antimicrobial compound':ti,ab,kw OR 'antimicrobial drug':ti,ab,kw OR 'antimicrobial factor':ti,ab,kw OR 'antiseptic':ti,ab,kw OR 'antiseptic agent':ti,ab,kw OR 'antiseptic cream':ti,ab,kw OR 'antiseptic foam':ti,ab,kw OR 'antiseptic soap':ti,ab,kw OR 'chemotherapeutic agent':ti,ab,kw OR 'chemotherapeutic drug':ti,ab,kw OR 'chemotherapeutica':ti,ab,kw OR 'microbiological agent':ti,ab,kw OR 'antiinfective agent':ti,ab,kw | 5972060 |
|  | 3 | 'smoking'/exp OR 'behavior, smoking':ti,ab,kw OR 'behaviour, smoking':ti,ab,kw OR 'reverse smoking':ti,ab,kw OR 'smoker':ti,ab,kw OR 'smokers':ti,ab,kw OR 'smoking behavior':ti,ab,kw OR 'smoking behaviour':ti,ab,kw OR 'tobacco smoking':ti,ab,kw OR 'smoking':ti,ab,kw | 722651 |
|  | 4 | 'air pollution'/exp OR 'aerial pollution':ab,ti,kw OR 'aerogenic pollution':ab,ti,kw OR 'air contamination':ab,ti,kw OR 'air pollutioning':ab,ti,kw OR 'air-borne pollution':ab,ti,kw OR 'airborne pollution':ab,ti,kw OR 'atmosphere pollution':ab,ti,kw OR 'atmospheric pollution':ab,ti,kw OR 'polluted air':ab,ti,kw OR 'polluted atmosphere':ab,ti,kw OR 'pollution, air':ab,ti,kw OR 'air pollution':ab,ti,kw | 264491 |
|  | 5 | 'helminthiasis'/exp OR 'helminth disease':ab,ti,kw OR 'helminth diseases':ab,ti,kw OR 'helminth infection':ab,ti,kw OR 'helminth infections':ab,ti,kw OR 'helminth infestation':ab,ti,kw OR 'helminth infestations':ab,ti,kw OR 'helminth parasitic infection':ab,ti,kw OR 'helminth parasitic infections':ab,ti,kw OR 'helminthiases':ab,ti,kw OR 'helminthic disease':ab,ti,kw OR 'helminthic infection':ab,ti,kw OR 'helminthic infections':ab,ti,kw OR 'helminthic infestation':ab,ti,kw OR 'helminthic infestations':ab,ti,kw OR 'helminthoses':ab,ti,kw OR 'helminthosis':ab,ti,kw OR 'infection with helminths':ab,ti,kw OR 'infection with parasitic worms':ab,ti,kw OR 'infection, helminthic':ab,ti,kw OR 'infection, worm':ab,ti,kw OR 'infections with helminths':ab,ti,kw OR 'parasitic worm infection':ab,ti,kw OR 'parasitic worm infestation':ab,ti,kw OR 'worm disease':ab,ti,kw OR 'worm diseases':ab,ti,kw OR 'worm infection':ab,ti,kw OR 'worm infections':ab,ti,kw OR 'worm infestation':ab,ti,kw OR 'worm infestations':ab,ti,kw OR 'helminthiasis':ab,ti,kw | 166528 |
|  | 6 | 'fluorocarbon'/exp OR 'fluor carbon':ab,ti,kw OR 'fluorocarbon compound':ab,ti,kw OR 'fluorocarbon derivative':ab,ti,kw OR 'fluorocarbon extract':ab,ti,kw OR 'fluorocarbon polymer':ab,ti,kw OR 'fluorocarbon polymers':ab,ti,kw OR 'fluorocarbon solvent':ab,ti,kw OR 'fluorocarbons':ab,ti,kw OR 'perfluorocarbon':ab,ti,kw OR 'perfluorocarbon compound':ab,ti,kw OR 'perfluorocarbon polymer':ab,ti,kw OR 'fluorocarbon':ab,ti,kw | 13115 |
|  | 7 | 'heavy metal'/exp OR 'metal, heavy':ab,ti,kw OR 'metals, heavy':ab,ti,kw OR 'heavy metal':ab,ti,kw | 268347 |
|  | 8 | 'pesticide'/exp OR 'agent, pesticide':ab,ti,kw OR 'pesticidal agent':ab,ti,kw OR 'pesticide agent':ab,ti,kw OR 'pesticide synergists':ab,ti,kw OR 'pesticides':ab,ti,kw OR 'scabicides/pediculicides':ab,ti,kw OR 'pesticide':ab,ti,kw | 510660 |
|  | 9 | 'environment'/exp OR 'environment determinant':ab,ti,kw OR 'environment':ab,ti,kw | 12708709 |
|  | 10 | 'light'/exp OR 'dim light':ab,ti,kw OR 'light beam':ab,ti,kw OR 'light diffusion':ab,ti,kw OR 'light flash':ab,ti,kw OR 'light induction':ab,ti,kw OR 'light radiation':ab,ti,kw OR 'light source':ab,ti,kw OR 'light wave':ab,ti,kw OR 'photoinduction':ab,ti,kw OR 'photon radiation':ab,ti,kw OR 'photoradiation':ab,ti,kw OR 'visible light':ab,ti,kw OR 'light':ab,ti,kw | 1318115 |
|  | 11 | 'fuel oil'/exp OR 'fuel oils':ab,ti,kw OR 'oil, fuel':ab,ti,kw OR 'fuel oil':ab,ti,kw | 2625 |
|  | 12 | 'pet animal'/exp OR 'animal companion':ab,ti,kw OR 'companion animal':ab,ti,kw OR 'pet animals':ab,ti,kw OR 'pet companion':ab,ti,kw OR 'pets':ab,ti,kw OR 'pet animal':ab,ti,kw | 21249 |
|  | 13 | 'meta analysis'/exp OR 'analysis, meta':ab,ti,kw OR 'meta-analysis':ab,ti,kw OR 'metaanalysis':ab,ti,kw OR 'meta analysis':ab,ti,kw OR 'meta analyses':ab,ti,kw OR 'meta-analyses':ab,ti,kw OR 'metaanalyses':ab,ti,kw | 546936 |
|  | 14 | #2 OR #3 OR #4 OR #5 OR #6 OR #7 OR #8 OR #9 OR #10 OR #11 OR #12 OR AND #13 | 293479 |
|  | 15 | #1 AND #14 | **1204** |
| **Web of Science** | 1 | TS=("Dermatitis, Atopic" OR "Atopic Dermatitis" OR "Eczema, Atopic" OR "Atopic Eczema" OR "Neurodermatitis, Atopic" OR "Atopic Neurodermatitis" OR "Neurodermatitis, Disseminated" OR "Disseminated Neurodermatitis" OR "Eczema, Infantile" OR "Infantile Eczema") | 36292 |
|  | 2 | TS=("Anti-Bacterial Agents" OR "Anti Bacterial Agents" OR "Anti-Bacterial Compounds" OR "Anti Bacterial Compounds" OR "Antibacterial Agents" OR "Anti-Bacterial Agent" OR "Anti Bacterial Agent" OR "Anti-Bacterial Compound" OR "Anti Bacterial Compound" OR "Antibacterial Agent" OR "Antibiotics" OR "Antibiotic" OR "Anti-Mycobacterial Agents" OR "Anti Mycobacterial Agents" OR "Antimycobacterial Agents" OR "Anti-Mycobacterial Agent" OR "Anti Mycobacterial Agent" OR "Antimycobacterial Agent" OR "Bacteriocidal Agents" OR "Bacteriocides" OR "Bacteriocidal Agent" OR "Bacteriocide") | 357781 |
|  | 3 | TS=("Smoking Behaviors" OR "Smoking Behavior" OR "Smoking Habit" OR "Smoking Habits" OR "Smoking") | 195575 |
|  | 4 | TS=("Air Pollution" OR "Air Quality") | 125043 |
|  | 5 | TS=("Helminthiases" OR "Nematomorpha Infection" OR "Nematomorpha Infections" OR "Helminthiasis") | 1415 |
|  | 6 | TS=("Perfluorocarbon" OR "Fluorocarbon" OR "Perfluorocarbons" OR "Polyfluorocarbons" OR "Per- and Polyfluoroalkyl Substances" OR "Per and Polyfluoroalkyl Substances" OR "PFAS" OR "Fluorinated Telomer Alcohols" OR "Fluoro-Telomer Alcohols" OR "Fluoro Telomer Alcohols" OR "Polyfluorinated Telomer Alcohols" OR "Perfluoroalkyl Carboxylates" OR "Perfluoroalkane Sulfonamides" OR "Perfluoroalkyl Ether Carboxylates" OR "Fluorotelomer Phosphate Esters" OR "Fluorocarbon Emulsions" OR "Fluorocarbon Emulsion" OR "Perfluoropolyether Carboxylic Acids" OR "Fluorocarbons") | 16615 |
|  | 7 | TS=("Heavy Metals" OR "Heavy Metal" OR "Metals, Heavy") | 171582 |
|  | 8 | TS=("Pesticide" OR "Pesticides") | 100456 |
|  | 9 | TS=("Environments" OR "Environmental Impact" OR "Environmental Impacts" OR "Environment") | 1756026 |
|  | 10 | TS=("Photoradiation" OR "Photoradiations" OR "Visible Light" OR "Visible Radiation" OR "Visible Radiations" OR "Light") | 1371003 |
|  | 11 | TS=("Fuel Oil" OR "Fuel Oils") | 3872 |
|  | 12 | TS=("Pet" OR "Companion Animals" OR "Companion Animal" OR "Companion Dogs" OR "Companion Dog" OR "Companion Cats" OR "Companion Cat" OR "Pets") | 188099 |
|  | 13 | TS=("meta-analysis" OR "meta analysis" OR "metaanalysis") | 499384 |
|  | 14 | #2 OR #3 OR #4 OR #5 OR #6 OR #7 OR #8 OR #9 OR #10 OR #11 OR #12 AND #13 | 44946 |
|  | 15 | #1 AND #14 | **155** |
| **Total** |  |  |  |

^1^ Two investigators (CSJ & ZSP) searched the online databases independently.

| **Supplementary Table 2**. Characteristics and quality assessment of the meta-analyses investigating environmental factors during childhood and Atopic dermatitis. | | | | | | |
| --- | --- | --- | --- | --- | --- | --- |
|  |  |  |  |  |  |  |
| Environmental factor | Author, year | Studies | Subjects | Random-effect model | | AMSTAR 2 |
|  |  | (n) | (n) | Relative risk and 95% CIs | P-value |  |
| Antibiotic | Duong et al., 2022 | 32 | 1,076,159 | OR 1.40(1.30-1.52) | P＜0.010 | Critically Low |
| Antibiotic | Ahmadizar et al., 2018 | 22 | NR | OR 1.26(1.15-1.37) | P=0.000 | Moderate |
| Maternal antibiotic | Zhong et al., 2021 | 7 | NR | OR 1.62(1.16-2.27) | P=0.004 | Moderate |
| Pesticides | Rodrigues et al., 2022 | 2 | NR | OR 2.19(0.51-9.36) | P=0.290 | Low |
| Active smoking | Kantor et al., 2016 | NR | 20 | OR 2.19(1.34-3.57) | P＜0.050 | Low |
| Active smoking | Saulyte et al., 2014 | 7 | NR | RR 1.36(1.17-1.46) | NR | Low |
| Passive smoking | Kantor et al., 2016 | NR | 66 | OR 1.15(1.01-1.30) | P＜0.050 | Low |
| Passive smoking | Saulyte et al., 2014 | 53 | NR | OR 1.06(1.01-1.11) | NR | Low |
| Prenatal active smoking | Kantor et al., 2016 | NR | NR | OR 1.06(0.80-1.40) | NR | Low |
| Prenatal active smoking | Zhou et al., 2021 | 13 | NR | OR 0.96(0.86-1.07) | P=0.017 | Low |
| Prenatal passive smoking | Zhou et al., 2021 | 4 | NR | OR 1.52(1.36-1.70) | P=0.233 | Low |
| Dog | Pelucchi et al., 2013 | 15 | NR | RR 0.72(0.61-0.85) | P=0.026 | Low |
| Cat | Pelucchi et al., 2013 | 13 | NR | RR 0.94(0.76-1.16) | P=0.010 | Low |
| Urban living | Shin et al., 2023 | NR | NR | OR 1.55(1.39-1.73) | NR | Moderate |
| Light at night | Deprato et al., 2024 | 4 | NR | OR 1.63(1.07-2.48) | P＜0.001 | Moderate |
| Indoor fuel | Lang et al., 2025 | 10 | NR | OR 1.16(1.05-1.28) | P=0.003 | Low |
| Trichuris trichiura | Agache et al., 2025 | 1 | 810 | HR 0.35(0.18-0.67) | P=0.950 | Moderate |
| Ascaris lumbricoides | Agache et al., 2025 | 1 | NR | HR 0.64(0.32-1.30) | NR | Moderate |
| hookworms | Agache et al., 2025 | 1 | 747 | HR 0.33(0.11-1.02) | NR | Moderate |
| Helminth | Arrais et al., 2022 | 11 | 23483 | RR 1.05(0.88-1.25) | NR | Low |
| PFNA | Hatem et al., 2025 | 5 | NR | OR 0.89(0.80-0.99) | P=0.911 | Moderate |
| PFNA | Luo et al., 2020 | 5 | 5276 | OR 0.89(0.80-0.99) | P=0.911 | Moderate |
| PFOS | Hatem et al., 2025 | 5 | NR | OR 0.91(0.81-1.02) | P=0.470 | Moderate |
| PFOA | Hatem et al., 2025 | 5 | NR | OR 0.99(0.88-1.01) | P=0.648 | Moderate |
| PFHxS | Hatem et al., 2025 | 5 | NR | OR 1.07(0.96-1.20) | P=0.202 | Moderate |
| Hg | Chen et al., 2024 | 8 | NR | OR 1.03(0.98-1.07) | NR | Moderate |
| Hg | Wang et al., 2022 | 5 | 3512 | OR 1.02(0.94-1.11) | P=0.000 | Moderate |
| Pb | Chen et al., 2024 | 8 | NR | OR 1.10(0.99-1.24) | NR | Moderate |
| Pb | Wang et al., 2022 | 4 | 3461 | OR 1.04(0.94-1.14) | P=0.080 | Moderate |
| Cd | Chen et al., 2024 | 8 | NR | OR 1.13(1.02-1.26) | NR | Moderate |
| Ni | Chen et al., 2024 | 8 | NR | OR 1.17(1.02-1.34) | NR | Moderate |
| Zn | Chen et al., 2024 | 8 | NR | OR 1.02(0.82-1.27) | NR | Moderate |
| Cu | Wang et al., 2022 | 1 | 2173 | OR 1.00(0.88-1.14) | NR | Moderate |
| PM2.5 | Ai et al., 2024 | 3 | NR | OR 1.34(1.10-1.63) | P=0.320 | Low |
| PM2.5（long-term） | Wang et al., 2022 | 2 | NR | RR 1.07(0.99-1.15) | P=0.870 | Moderate |
| PM10 | Ai et al., 2024 | 8 | NR | OR 1.00(0.91-1.10) | P＜0.010 | Low |
| PM10（long-term） | Wang et al., 2022 | 3 | NR | RR 1.05(1.01-1.09) | P=0.733 | Moderate |
| NO2 | Ai et al., 2024 | 8 | NR | OR 1.11(1.05-1.16) | P=0.310 | Low |
| NO2（long-term） | Wang et al., 2022 | 5 | NR | RR 1.02(0.99-1.06) | P=0.062 | Moderate |
| SO2（long-term） | Wang et al., 2022 | 2 | NR | RR 1.14(0.83-1.56) | P=0.046 | Moderate |
| CO（long-term） | Wang et al., 2022 | 3 | NR | RR 1.01(1.01-1.01) | P=0.194 | Moderate |
| O3 （long-term） | Wang et al., 2022 | 4 | NR | RR 1.00(0.99-1.02) | P=0.316 | Moderate |
| NO2（short-term） | Wang et al., 2022 | 2 | NR | RR 1.00(0.99-1.00) | P=0.897 | Moderate |
| O3 （short-term） | Wang et al., 2022 | 3 | NR | RR 1.03(0.99-1.08) | P=0.004 | Moderate |
|  |  |  |  |  |  |  |
| **Abbreviations:** OR, odds ratio; HR, hazard ratio; RR, risk ratio; NR, not reported. | | | | | | |

| **Supplementary Table 3**. Characteristics and quality assessment of the meta-analyses investigating environmental factors during adulthood and Atopic dermatitis. | | | | | | |
| --- | --- | --- | --- | --- | --- | --- |
|  |  |  |  |  |  |  |
| Environmental factor | Author, year | Studies | Subjects | Random-effect model | | AMSTAR 2 |
|  |  | (n) | (n) | Relative risk and 95% CIs | P-value |  |
| Light at night | Deprato et al., 2024 | 8 | NR | OR 1.30(1.03-1.63) | P＜0.001 | Moderate |
| Active smoking | Kantor et al., 2016 | NR | 20 | OR1.30(1.06-1.59) | P＜0.050 | Low |
| Active smoking | Saulyte et al., 2014 | 23 | NR | RR 1.14(1.07-1.22) | NR | Low |
| Passive smoking | Kantor et al., 2016 | NR | 66 | OR 3.62(1.71-7.69) | P＜0.050 | Low |
| Passive smoking | Saulyte et al., 2014 | 4 | NR | RR 1.26(1.02-1.55) | NR | Low |
| Urban living | Shin et al., 2023 | NR | NR | OR 1.29(0.99-1.67) | NR | Moderate |
| Helminth | Arrais et al., 2022 | 11 | 23483 | RR 1.05(0.88-1.25) | NR | Low |
| PM2.5 | Hsiao et al., 2022 | 2 | NR | OR 2.30(1.25-4.25) | P=0.773 | Moderate |
| PM2.5 | Wang et al., 2022 | 2 | NR | RR 1.18(0.77-1.81) | P=0.014 | Moderate |
| NO2 | Arrais et al., 2022 | 2 | NR | OR 1.30(1.04-1.61) | P=0.853 | Moderate |
| NO2 | Wang et al., 2022 | 2 | NR | RR 1.10(0.97-1.24) | P=0.835 | Moderate |
|  |  |  |  |  |  |  |
| **Abbreviations:** OR, odds ratio; HR, hazard ratio; RR, risk ratio; NR, not reported. | | | | | | |

| **Supplementary Table 4**. Characteristics and quality assessment of the meta-analyses investigating environmental factors across all age groups and Atopic dermatitis. | | | | | | |
| --- | --- | --- | --- | --- | --- | --- |
|  |  |  |  |  |  |  |
| Environmental factor | Author, year | Studies | Subjects | Random-effect model | | AMSTAR 2 |
|  |  | (n) | (n) | Relative risk and 95% CIs | P-value |  |
| Active smoking | Kantor et al., 2016 | NR | 20 | OR1.87(1.32-2.63) | P＜0.050 | Low |
| Active smoking | Saulyte et al., 2014 | 33 | NR | RR 1.21(1.14-1.29) | NR | Low |
| Passive smoking | Kantor et al., 2016 | NR | 66 | OR 1.18(1.01-1.38) | P＜0.050 | Low |
| Passive smoking | Saulyte et al., 2014 | 58 | NR | RR 1.02(0.92-1.15) | NR | Low |
| Urban living | Shin et al., 2023 | 43 | 1728855 | OR 1.56(1.43-1.71) | P＜0.001 | Moderate |
| Helminth | Arrais et al., 2022 | 11 | 20860 | RR 1.04(0.87-1.25) | NR | Low |
| PM10（long-term） | Wang et al., 2022 | 2 | NR | RR 1.58(1.33-1.89) | NR | Moderate |
| PM10（short-term） | Wang et al., 2022 | 8 | NR | RR 1.01(1.00-1.01) | NR | Moderate |
| NO2（short-term） | Wang et al., 2022 | 7 | NR | RR 1.01(1.01-1.01) | NR | Moderate |
| SO2（short-term） | Wang et al., 2022 | 2 | NR | RR 1.01(1.00-1.02) | NR | Moderate |
|  |  |  |  |  |  |  |
| Abbreviations: OR, odds ratio; HR, hazard ratio; RR, risk ratio; NR, not reported. | | | | | | |

| **Supplementary Table 5.** Quality assessment of included meta-analyses with AMSTAR 2. | | | | | | | | | | | | | | | | | |
| --- | --- | --- | --- | --- | --- | --- | --- | --- | --- | --- | --- | --- | --- | --- | --- | --- | --- |
| **ITEMS** | | | | | | | | | | | | | | | | | **Final rating** |
|  |  |  |  |  |  |  |  |  |  |  |  |  |  |  |  |  |  |
|  | 1 | 2* | 3 | 4* | 5 | 6 | 7* | 8 | 9* | 10 | 11* | 12 | 13* | 14 | 15* | 16 |  |
| Duong et al., 2022 | Y | N | N | PY | N | N | N | Y | Y | N | Y | Y | Y | Y | N | Y | Critically Low |
| Ahmadizar et al., 2018 | Y | N | Y | PY | N | N | N | Y | PY | N | Y | N | Y | Y | Y | Y | Moderate |
| Zhong et al., 2021 | Y | Y | Y | Y | Y | Y | N | Y | Y | N | Y | Y | Y | Y | Y | Y | Moderate |
| Rodrigues et al., 2022 | Y | Y | Y | Y | PY | N | N | Y | Y | N | Y | N | PY | Y | N | Y | Low |
| Kantor et al., 2016 | Y | N | Y | Y | Y | Y | N | Y | Y | N | Y | PY | Y | Y | Y | Y | Low |
| Saulyte et al., 2014 | Y | N | Y | Y | Y | Y | N | Y | PY | Y | Y | Y | Y | Y | Y | Y | Low |
| Zhou et al., 2021 | Y | N | Y | Y | Y | Y | N | Y | Y | N | Y | Y | Y | Y | N | Y | Low |
| Pelucchi et al., 2013 | Y | Y | Y | Y | Y | Y | N | Y | N | N | Y | Y | Y | Y | N | Y | Low |
| Shin et al., 2023 | Y | Y | Y | Y | Y | Y | N | Y | Y | N | Y | Y | Y | Y | Y | Y | Moderate |
| Deprato et al., 2024 | Y | Y | Y | Y | PY | N | N | Y | Y | N | Y | Y | Y | Y | Y | Y | Moderate |
| Lang et al., 2025 | Y | Y | Y | Y | Y | Y | N | Y | Y | N | Y | Y | Y | Y | N | Y | Low |
| Agache et al., 2025 | Y | N | Y | Y | Y | Y | Y | N | Y | Y | Y | Y | Y | Y | Y | Y | Moderate |
| Hatem et al., 2025 | Y | N | Y | Y | Y | Y | Y | Y | Y | N | Y | Y | Y | Y | Y | Y | Moderate |
| Luo et al., 2020 | Y | Y | Y | Y | N | N | N | Y | Y | N | Y | N | Y | Y | Y | Y | Moderate |
| Arrais et al., 2022 | Y | Y | Y | Y | Y | Y | N | Y | Y | N | Y | PY | Y | Y | Y | Y | Low |
| Chen et al., 2024 | Y | Y | Y | Y | PY | N | N | Y | Y | N | Y | N | Y | Y | Y | Y | Moderate |
| Wang et al., 2022 | Y | Y | Y | Y | Y | N | N | Y | Y | N | Y | Y | Y | Y | Y | Y | Moderate |
| Ai et al., 2024 | Y | Y | Y | Y | PY | N | N | Y | Y | N | Y | Y | PY | Y | Y | Y | Low |
| Wang et al., 2022 | Y | N | Y | Y | PY | N | N | Y | Y | N | Y | Y | Y | Y | Y | Y | Moderate |
| Hsiao et al., 2022 | Y | Y | Y | Y | N | N | N | Y | Y | N | Y | Y | Y | Y | PY | Y | Moderate |

**Abbreviations**: Y = Yes; N = No; PY = Partial Yes.

The 16 categories correspond to the domains of methodological quality assessment based on the AMSTAR 2 tool, including protocol registration, literature search comprehensiveness, risk of bias assessment, and appropriateness of meta-analytic methods.

**Quality rating**: The overall rating (High, Moderate, Low, or Critically Low) was determined based on the presence of critical and non-critical weaknesses, in accordance with AMSTAR 2 guidance. Reviews with no or one non-critical weakness were rated as High; those with more than one non-critical weakness were rated as Moderate; those with one critical flaw were rated as Low; and those with more than one critical flaw were rated as Critically Low. **A detailed description of each item is provided in the AMSTAR 2 checklist.**

| **Supplementary Table 6**. GRADE-informed certainty of evidence for environmental exposures associated with atopic dermatitis across different age groups. | | | | | | | | | |
| --- | --- | --- | --- | --- | --- | --- | --- | --- | --- |
|  |  |  |  |  |  |  |  |  |  |
| Population | Exposure | Effect estimate (95% CI) | Risk of bias | Inconsistency | Indirectness | Imprecision | Publication bias | Dose–response relationship | Overall certainty |
| Children | Antibiotics | ORs ranged from 1.26 (1.15–1.37) to 1.62 (1.16–2.27) | Serious | Not serious | Not serious | Not serious | Suspected | Not reported | Low |
| Children | Helminth infections | HRs ranged from 0.33 (0.11–1.02) to 0.64 (0.32–1.30) | Serious | Serious | Not serious | Serious | Suspected | Not reported | Very low |
| Children | PFAS | ORs ranged from 0.89 (0.80–0.99) to 1.07 (0.96–1.20) | Serious | Serious | Not serious | Not serious | Suspected | Inconsistent | Very low |
| Children | Heavy metals | ORs ranged from 1.00 (0.88–1.14) to 1.17 (1.02–1.34) | Serious | Serious | Not serious | Not serious | Suspected | Limited evidence | Very low |
| Children | Air pollution | PM₂.₅: 1.34 (1.10–1.63) | Serious | Not serious | Not serious | Not serious | Suspected | Evidence present for PM₂.₅ and NO₂; not observed for PM₁₀ | Low |
|  |  | NO₂: 1.11 (1.05–1.16) |  |  |  |  |  |  |  |
|  |  | PM₁₀: 1.00 (0.91–1.10) |  |  |  |  |  |  |  |
| Children | Smoking | ORs ranged from 0.96 (0.86–1.07) to 2.19 (1.34–3.57) | Serious | Serious | Not serious | Not serious | Suspected | No clear dose–response relationship | Very low |
| Children | Pet exposure | Dog: RR 0.72 (0.61–0.85) | Serious | Serious | Not serious | Not serious | Suspected | Not reported | Very low |
|  |  | Cat: RR 0.94 (0.76–1.16) |  |  |  |  |  |  |  |
| Children | Urban living | OR 1.55 (1.39–1.73) | Serious | Not applicable | Not serious | Serious | Suspected | Not reported | Very low |
| Children | Light at night | OR 1.63 (1.07–2.48) | Serious | Not applicable | Not serious | Serious | Suspected | Not reported | Very low |
| Children | Indoor fuel use | OR 1.16 (1.05–1.28) | Serious | Not applicable | Not serious | Serious | Suspected | Not reported | Very low |
| Adults | Light at night | OR 1.30 (1.03–1.63) | Serious | Not applicable | Not serious | Serious | Suspected | Not reported | Very low |
| Adults | Smoking | Active: OR 1.30 (1.06–1.59) / RR 1.14 (1.07–1.22) | Serious | Serious | Not serious | Not serious | Suspected | No clear dose–response relationship | Very low |
|  |  | Passive: OR 3.62 (1.71–7.69) / RR 1.26 (1.02–1.55) |  |  |  |  |  |  |  |
| Adults | Urban living | OR 1.29 (0.99–1.67) | Serious | Not applicable | Not serious | Serious | Suspected | Not reported | Very low |
| Adults | Helminth infections | RR 1.05 (0.88–1.25) | Serious | Not applicable | Not serious | Serious | Suspected | Not reported | Very low |
| Adults | Air pollution | PM₂.₅: OR 2.30 (1.25–4.25) / RR 1.18 (0.77–1.81) | Serious | Serious | Not serious | Serious | Suspected | Limited evidence | Very low |
|  |  | NO₂: OR 1.30 (1.04–1.61) / RR 1.10 (0.97–1.24) |  |  |  |  |  |  |  |
| Mixed-age populations | Smoking | Active: OR 1.87 (1.32–2.63) / RR 1.21 (1.14–1.29) | Serious | Serious | Not serious | Not serious | Suspected | No clear dose–response relationship | Very low |
|  |  | Passive: OR 1.18 (1.01–1.38) / RR 1.02 (0.92–1.15) |  |  |  |  |  |  |  |
| Mixed-age populations | Urban living | OR 1.56 (1.43–1.71) | Serious | Not applicable | Not serious | Not serious | Suspected | Not reported | Low |
| Mixed-age populations | Helminth infections | RR 1.04 (0.87–1.25) | Serious | Not applicable | Not serious | Serious | Suspected | Not reported | Very low |
| Mixed-age populations | Air pollution | PM₁₀ (long-term): RR 1.58 (1.33–1.89) | Serious | Serious | Serious | Not serious | Suspected | Evidence present, but limited | Very low |

| **Note:** A simplified GRADE-informed framework was used to assess the certainty of evidence, considering risk of bias, inconsistency, indirectness, imprecision, and publication bias. Dose–response relationships were qualitatively evaluated where reported. Certainty was assessed at the exposure level rather than at the individual study level. As all included evidence was derived from observational studies, the initial certainty level was considered low and was further downgraded based on the above domains. |
| --- |
|  |
|  |
|  |
|  |
| **Abbreviations:** CI, confidence interval; OR, odds ratio; HR, hazard ratio; RR, risk ratio. |

| **Supplementary Table 7**. List of abbreviations used in the manuscript | |
| --- | --- |
| **Abbreviation** | **Full term** |
| AD | Atopic dermatitis |
| MeSH | Medical Subject Headings |
| OR | Odds ratio |
| RR | Relative risk |
| HR | Hazard ratio |
| CI | Confidence interval |
| PFAS | Per- and polyfluoroalkyl substances |
| AMSTAR 2 | A Measurement Tool to Assess Systematic Reviews 2 |
| I² | I-squared statistic |
| PM₂.₅ | Particulate matter with an aerodynamic diameter ≤2.5 μm |
| PM₁₀ | Particulate matter with an aerodynamic diameter ≤10 μm |
| NO₂ | Nitrogen dioxide |
| SO₂ | Sulfur dioxide |
| PFNA | Perfluorononanoic acid |
| IgE | Immunoglobulin E |
